# Dissociating Mechanisms That Underlie Seasonal and Developmental Programs for the Neuroendocrine Control of Physiology in Birds

**DOI:** 10.1523/ENEURO.0154-23.2023

**Published:** 2024-04-08

**Authors:** Timothy Adam Liddle, Gaurav Majumdar, Calum Stewart, Maureen M. Bain, Tyler John Stevenson

**Affiliations:** ^1^Laboratory of Seasonal Biology, School of Biodiversity, One Health, and Veterinary Medicine, University of Glasgow, Glasgow, United Kingdom; ^2^Department of Zoology, University of Allahabad, Allahabad, India

**Keywords:** *Coturnix japonica*, MBH, Oxford Nanopore RNA sequencing, photoperiod, pituitary gland, seasonality

## Abstract

Long-term programmed rheostatic changes in physiology are essential for animal fitness. Hypothalamic nuclei and the pituitary gland govern key developmental and seasonal transitions in reproduction. The aim of this study was to identify the molecular substrates that are common and unique to developmental and seasonal timing. Adult and juvenile quail were collected from reproductively mature and immature states, and key molecular targets were examined in the mediobasal hypothalamus (MBH) and pituitary gland. qRT-PCR assays established deiodinase type 2 (*DIO2*) and type 3 (*DIO3*) expression in adults changed with photoperiod manipulations. However, *DIO2* and *DIO3* remain constitutively expressed in juveniles. Pituitary gland transcriptome analyses established that 340 transcripts were differentially expressed across seasonal photoperiod programs and 1,189 transcripts displayed age-dependent variation in expression. Prolactin (*PRL*) and follicle-stimulating hormone subunit beta (*FSHβ*) are molecular markers of seasonal programs and are significantly upregulated in long photoperiod conditions. Growth hormone expression was significantly upregulated in juvenile quail, regardless of photoperiodic condition. These findings indicate that a level of cell autonomy in the pituitary gland governs seasonal and developmental programs in physiology. Overall, this paper yields novel insights into the molecular mechanisms that govern developmental programs and adult brain plasticity.

## Significance Statement

Seasonal physiology is pervasive in the animal kingdom. While much is known regarding how the brain perceives annual changes in daylength (also referred to as photoperiod) and dynamics of the neuroendocrine control of seasonal physiology in adult animals, studies in juveniles are limited. Here, we assess genome-wide and targeted transcriptomic changes in the pituitary gland, a key brain region connecting photoreception with physiological plasticity in adult and juvenile Japanese quail. The analyses identified several novel transcripts that are correlated with photoperiod- and developmental programs in seasonal physiology. The findings demonstrate a level of pituitary gland cell specificity for the regulation of both development and reproductive fitness that is dependent on both age and experienced photoperiod.

## Introduction

Seasonal and developmental programs in the neuroendocrine control of reproductive physiology in mammals and birds are well characterized (Stevenson et al., 2012a,b; Plant, 2015; Stevenson et al., 2017). However, very few studies have directly compared the similarities and differences in how the hypothalamus governs these long-term changes in physiology. Animals in temperate regions experience variable environmental conditions within and across seasons, including changes in daylength, ambient temperature, and availability of food (Sharp, 1996; Hau, 2001). The annual change in daylength, referred to as photoperiod, is a powerful signal that animals use as a predictive cue to anticipate environmental conditions ideal for breeding (Bradshaw and Holzapfel, 2007; Wood and Loudon, 2014; Payton et al., 2017; Stevenson et al., 2022). In most mammals, the nocturnal duration of melatonin secretion from the pineal gland provides a physiological code of photoperiod and drives many molecular, cellular, and morphological changes in the median eminence and pituitary gland (Wood and Loudon, 2014; Stevenson et al., 2022). Conversely, birds have photoreceptors located deep in the hypothalamus that directly detect light stimulation to drive photoperiod-induced changes in seasonal physiology (Stevenson and Ball, 2012; Pérez et al., 2019, 2023; Liddle et al., 2022). Despite these markedly different neuroendocrine control mechanisms, the pituitary gland is a conserved anatomical structure that provides the essential gating mechanism to permit and inhibit communication from the hypothalamus to peripheral tissues (e.g., gonads).

Juvenile animals have a heightened sensitivity to the effects of photoperiod on reproductive physiology. Siberian hamsters raised in long summer-like photoperiods initiate puberty at approximately postnatal day 21 (Spears et al., 1990). Transfer of hamsters to short winter-like photoperiods induces a rapid inhibition of gonadal development resulting in gonadal involution in <7 d (Prendergast et al., 2004). A single subcutaneous injection of melatonin to weaned hamsters (i.e., postnatal day18) is sufficient to prevent gonadal growth, despite being housed in stimulatory long photoperiods (Prendergast et al., 2013). Similarly, Japanese quail reared in long stimulatory photoperiods reach sexual maturity by 28–35 d posthatch (Follett, 1976). Birds that are transferred to shorter photoperiods (e.g., 12 light, 12 dark) delay gonadal growth by up to 2 weeks (Follett and Maung, 1978), and photoperiods <12 h light completely prevent reproductive maturation (Follett and Sharp, 1969; Abdelnabi and Ottinger, 2003).

Only a few studies have examined how photoperiod manipulations early in development impact the neuroendocrine regulation of reproductive physiology. In adult birds and mammals, the expression of deiodinase enzymes, deiodinase type 2 (*DIO2*) and deiodinase type 3 (*DIO3*), is a molecular signature of a photoperiodic state (Nakane and Yoshimura, 2014; Rani and Kumar, 2014). Long photoperiods increase *DIO2* expression, which catalyzes the conversion of inactive thyroxine (T4) into active triiodothyronine (T3), and short photoperiods initiate the reversal by stimulating *DIO3* expression and reducing hypothalamic triiodothyronine (Yoshimura et al., 2003; Barrett et al., 2007). In juvenile Siberian hamsters, transfer to short photoperiods or subcutaneous injections of melatonin rapidly increases *DIO3* expression in the mediobasal hypothalamus (MBH; Prendergast et al., 2013; Sáenz de Miera et al., 2017). Whether similar rapid changes occur in juvenile birds is currently unknown. Resolving whether *DIO3* or *DIO2* display an augmented sensitivity to photoperiodic cues early in development is important to develop our understanding of conserved neuroendocrine pathways that underlie photoperiod and developmental programming of seasonal physiology.

Seasonal and reproductive maturation requires the release of gonadotropin-releasing hormone (*GnRH*) into the hypophyseal portal system, allowing for transport into the pituitary gland where GnRH acts on gonadotrophs to stimulate the release of luteinizing hormone (*LH*) and follicle-stimulating hormone (*FSH*; Stevenson et al., 2012a,b). Therefore, the pituitary gland can be viewed as the final mediator between seasonal and developmental programming of seasonal physiology. The objectives of this study were twofold. First, the expression profiles of *DIO2* and *DIO3* in the MBH tissue were examined in adult and juvenile Japanese quail held in long or short photoperiods. The findings indicate neither *DIO2* nor *DIO3* show a similar pattern in adults compared with juveniles suggesting a clear dissociation between developmental programming and seasonal photoperiodic regulation of deiodinase expression. The second objective was to identify seasonal and developmental programmed changes in the pituitary gland. Oxford Nanopore RNA sequencing was conducted to produce a pituitary transcriptome and enable a comprehensive overview of age- and light-dependent molecular changes. The data described herein suggest cellular specificity in how the pituitary gland controls long-term programs in physiology.

## Materials and Methods

### Animals and ethical permissions

Japanese quail were purchased from MoonRidge Farms, Exeter. Five-week-old male quail (*n* = 12) were housed on a 16 L:8 D (L, light; D, dark) light schedule. All birds were provided Farmgate layers pellets and tap water *ad libitum*. Two-day-old Japanese quail eggs (*n* = 40) were placed in an incubator (Brinsea OVA Easy Advance 380; temperature, 37.5°C; relative humidity, 50–60%) until hatching (16–18 d). Hatched chicks (*n* = 8 males, *n* = 3 females) were moved to quail pens provided with heat lamps (37.5°C) and provided blended Heygates Superstarter Crumb and tap water *ad libitum*. Animal research was conducted according to the ARRIVE guidelines and Home Office–approved project and establishment licenses. All animal procedures were performed in accordance with the relevant establishment's animal care committee regulations.

### Experimental design

Five-week-old birds were acclimated to a long photoperiod for 2 weeks. Then birds were placed on nonstimulatory photoperiods to induce gonadal involution and collected in short days (8 L) after 8 weeks. A subset of the short-day birds (*n* = 6) was humanely killed using cervical dislocation followed by decapitation. Another group (*n* = 6) was placed on stimulatory photoperiods to induce gonadal growth and collected in a long photoperiod after 8 weeks. Newly hatched juveniles were acclimated to a long photoperiod for 5 d. Birds were divided into two groups pseudorandomly assigned in either a long (16 L, *n* = 6) or short photoperiod (8 L, *n* = 5) for 5 d. The 16 L juvenile group contained four males and two females, whereas the 8 L juvenile group contained four males and one female. On posthatch day 10, chicks were killed by cervical dislocation followed by decapitation. For all birds, brain and pituitary tissues were frozen on dry ice and stored at −70°C. During collections, the pituitary stalk was severed, leaving the pars tuberalis attached to the MBH. Pituitary tissue including the pars distalis, retained for both qRT-PCR analyses and sequencing, was dissected from the sella turcica. Testis length was measured using calipers to the nearest 0.01 mm. Ovary length of the three female chicks was measured to confirm the photoperiod state. Birds were assigned a fat score from 0 to 5 based on a scale established in white-crowned sparrows (Wingfield and Farner, 1978).

### Whole-brain dissections

To isolate the MBH/hypothalamic regions, a brain matrix was used within a cryostat chamber. Previously published anatomical coordinates acted as guidance for dissection (Nakao et al., 2008; Stevenson et al., 2012a,b). Brains were positioned ventral side up and oriented with the caudal direction facing upward. The MBH was dissected using a brain matrix. First, a 3 mm coronal section from the optic chiasm in a posterior direction was performed. Then 2 mm lateral cuts and a 2 mm dorsal cut were conducted to isolate the MBH. This dissection protocol reliably isolates the MBH in Japanese quail (Majumdar et al., 2023). Brains were then returned to −70°C.

### RNA extraction and cDNA synthesis

For all samples, RNA was extracted using TRIzol reagent following the manufacturer’s protocol. RNA was then purified using an RNEasy MinElute cleanup kit (Qiagen). RNA quality and purity were measured with NanoDrop ND-100 (NanoDrop Technologies). Both pituitary gland and hypothalamic RNA were retained for cDNA synthesis and qRT-PCR analyses; however, some pituitary gland RNA were also retained for direct use in RNA sequencing. cDNA was synthesized from TRIzol-extracted RNA using a reaction mixture containing 4 μl ∼50 ng/μl total RNA (∼200 ng total), 2 μl 5× first-strand buffer (Thermo Fisher Scientific), 1 μl DTT (10 mM), 0.2 μl 20 mM random primers (Promega), 0.2 μl 20 mM dNTP mix (Thermo Fisher Scientific), 0.26 μl RNasin ribonuclease inhibitor (Promega), 0.26 μl SuperScript III reverse transcriptase (Thermo Fisher Scientific), and 2.08 μl RNase-free water. The reaction mixture was incubated at 42°C for 1 min followed by 50°C for 1 h. cDNA mixtures were diluted in LOTE buffer (3 mM Tris-HCl (Thermo Fisher Scientific) and 0.2 mM EDTA (Sigma-Aldrich) before storage at −20°C.

### qRT-PCR procedure

Transcription levels of target genes were quantified using SYBR Green Real-Time PCR master mix (Thermo Fisher Scientific) and specific primer sequences for amplification (Table 1). cDNA samples were amplified using an Agilent Stratagene Mx3000p under the following conditions: (1) denaturing: 95°C for 10 min; (2) cycling: 45 times through a 95°C denature for 30 s, an annealing temperature for 1 min (primer specific), and an extension of 72°C for 1 min. A melting curve assay after the qRT-PCR amplification consisted of increasing from 55°C, held for 30 s, to 95°C. PCR Miner was used to quantify cycling times, reaction efficiency, and sample variability (Zhao and Fernald, 2005). ß-Actin was chosen as the reference gene for quantification of targeted transcript expression. The standard ΔΔCt method was used to produce fold change in gene expression (2^−ΔΔCt^), with the adult 8 L group acting as the reference for the calculation of the ΔΔ value.

**Table 1.** Targeted qRT-PCR primer sequences and specifications, including forward and reverse primer sequences, annealing temperature, melting temperature, and expected product sizes for *DIO2*, *DIO3*, *OPN5*, *LHβ*, *FSHβ*, *PRL*, and *GH*

Gene	Primer	Annealing temperature (°C)	Melting temperature (°C)	Length (bp)
*DIO2*	CGCCTACAAGCAGGTCAAAC	60	82	242
	CACACTTGCCACCAACACTCTT			
*DIO3*	AGGCTCTCTTCCTTCGGGAT	60	83	180
	TAGCACTTGCTAGGCAGCAC			
*OPN5*	ATGGCATCAGACTGCAACTCC	60	84	499
	AAGGAACAGTAGCCCAGAACG			
*LHΒ*	TTTACCGCAGCCCTTTGGGT	60	87	125
	AGAGCCACGGGTAGGATGACTTT			
*FSHΒ*	CTGCGGTGACCATCCTGAATCTTT	62	85	396
	GCTTCCATTGTGACTGAAGGAGCA			
*PRL*	AATGAAACCCCGACCCTGAG	60	79	630
	CCCCTAGTGCAACTTGAGACC			
*GH*	GCTGCCGAGACATACAAAGAG	60	81	109
	GAGCTGGGATGGTTTCTGAG			
ß-actin	AATCAAGATCATTGCCCCAC	60	84	114
	TAAGACTGCTGCTGACACC			

### Oxford Nanopore RNA sequencing procedure

TRIzol-extracted RNA was sequenced using a GridION (Oxford Nanopore Technologies) after processing using the protocol outlined in the Oxford Nanopore Technologies PCR-cDNA sequencing barcoding kit (SQK-PCB109). Raw Fast5 reads were base called and demultiplexed using the guppy base caller before removing adapters from reads and filtering long reads (>25 bp) using Porechop and Filtlong, respectively. The parameters for each sequencing assay included running at a voltage of −180 mV for 72 h. Gene transcripts were aligned to a reference *Coturnix japonica* genome (https://www.ncbi.nlm.nih.gov/datasets/genome/GCF_001577835.1/) before using Salmon to quantify transcript expression levels. EdgeR was used to identify differentially expressed genes based on their significance value (*p* < 0.01). For analyses comparing all groups, the adult 8 L group was chosen as the reference for comparison.

### Gene ontology

Functional annotation of transcripts identified by Oxford Nanopore RNA sequencing was performed using the Database for Annotation, Visualization and Integrated Discovery (DAVID, Sherman et al., 2022). Significantly differentially expressed transcripts across both photoperiod and age were categorized using the functional annotation chart tool.

### Statistical analyses and plots

All raw data are provided in extended data (Extended Data Table 2-1). Two-way ANOVA was conducted on testis length, fat score, and hypothalamic *DIO2* and *DIO3* expression. EdgeR was used to determine statistically significant differentially expressed transcripts in the pituitary gland (Robinson et al., 2010; McCarthy et al., 2012; Chen et al., 2016). For the testis length and fat score measures, the three juvenile females were omitted from the analyses. All plots were created in RStudio (R Core Team, 2013; RStudio Team, 2015) using the ggplot2 package, and figures were created using Adobe Illustrator. For all statistical analyses, a *p*-value of <0.05 [and a false discovery rate (FDR) < 0.2, where applicable] was defined as the statistical cutoff for significance. This FDR significance threshold was determined based on the knowledge that *FSHβ* is significantly upregulated in long day adults.

## Results

### Short photoperiods induce gonadal and adipose involution in adult and juvenile quail

**Figure 1.**
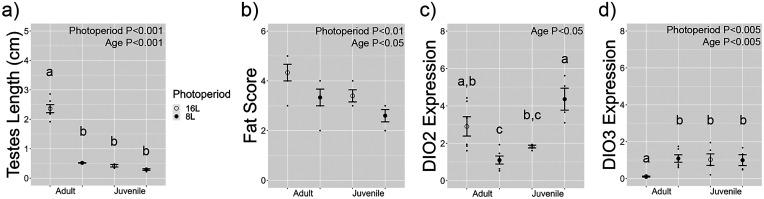
Long photoperiods induce physiological and hypothalamic neuroendocrine change associated with reproduction in adult and juvenile Japanese quail. ***a***, Measurements of testis length showed a significant interaction between photoperiod and age. ***b***, Fat score ratings, as established by Wingfield and Farner, were overall higher in adults and in 16L photoperiod conditions. ***c***, qRT-PCR analyses show a significant photoperiod by age interaction on *DIO2* expression in the MBH. ***d***, A significant photoperiod by age interaction was also found in MBH *DIO3* expression. Physiological and transcriptomic data from qRT-PCR were analyzed by two-way ANOVA and Tukey's HSD with an *α* value of *p* = 0.05. The letters above each group indicate pairwise comparisons by Tukey's HSD where appropriate.

Two-way ANOVA identified that short photoperiod significantly reduced testis length in adult and juvenile quail (Fig. 1*a*, Table 2). The accompanying table includes *F*- and *P*- statistics for all two-way ANOVA analyses in both Figures 1 and 3. Adult quail had significantly larger testes compared with juveniles. There was a significant interaction suggesting photoperiod effects were larger in adults compared with juvenile birds. Two females in long photoperiod had large ovaries (0.49 and 0.57 cm), and the single female in short photoperiod had a regressed ovary (0.13 cm); however, it was not possible to collect data regarding follicular development. Short photoperiod also reduced adipose tissue indicated by lower fat scores compared with birds in long photoperiod (Fig. 1*b*). Juvenile quail had lower fat scores compared with adult birds. There was no significant photoperiod by development interaction. All three juvenile females, omitted from the fat score analyses, had a fat score of 3.

**Table 2.** Summary of two-way ANOVA analyses associated with Figures 1 and 3, including *F*- and *P*-statistics associated with the main effects of photoperiod and age, and their interaction, on testis length, fat score, and the expression of *DIO2*, *DIO3*, *FSHβ*, *LHβ*, *PRL*, *GH*, and *OPN5E*

	Photoperiod	Age	Photoperiod:age
Testis length	*F*_(1,15)_ = 198.46, *p* = 4.69 × 10^−10^***	*F*_(1,15)_ = 112.75, *p* = 2.26 × 10^−8^***	*F*_(1,15)_ = 81.17, *p* = 1.94 × 10^−7^***
Fat score	*F*_(1,16)_ = 9.23, *p* = 7.83 × 10^−3^**	*F*_(1,16)_ = 6.15, *p* = 2.46 × 10^−2^*	*F*_(1,16)_ = 0.00, *p* = 1.00 n.s.
*DIO2*	*F*_(1,16)_ = 0.77, *p* = 3.94 × 10^−1^ n.s.	*F*_(1,16)_ = 6.94, *p* = 1.81 × 10^−2^*	*F*_(1,16)_ = 22.12, *p* = 2.4 × 10^−4^***
*DIO3*	*F*_(1,17)_ = 10.78, *p* = 4.38 × 10^−3^**	*F*_(1,17)_ = 13.40, *p* = 1.94 × 10^−3^**	*F*_(1,17)_ = 15.30, *p* = 1.12 × 10^−3^**
*FSHβ*	*F*_(1,17)_ = 17.69, *p* = 5.95 × 10^−4^***	*F*_(1,17)_ = 29.54, *p* = 4.45 × 10^−5^***	*F*_(1,17)_ = 0.83, *p* = 3.75 × 10^−1^ n.s.
*LHβ*	*F*_(1,17)_ = 9.47, *p* = 6.83 × 10^−3^**	*F*_(1,17)_ = 74.37, *p* = 1.29 × 10^−7^***	*F*_(1,17)_ = 4.68, *p* = 4.51 × 10^−2^*
*PRL*	*F*_(1,17)_ = 9.27, *p* = 7.34 × 10^−3^**	*F*_(1,17)_ = 14.64, *p* = 1.35 × 10^−3^**	*F*_(1,17)_ = 1.92, *p* = 1.83 × 10^−1^ n.s.
*GH*	*F*_(1,16)_ = 3.46, *p* = 8.14 × 10^−2^ n.s.	*F*_(1,16)_ = 21.48, *p* = 2.75 × 10^−4^***	*F*_(1,16)_ = 5.67, *p* = 3.00 × 10^−2^*
*OPN5*	*F*_(1,16)_ = 17.01, *p* = 7.96 × 10^−4^***	*F*_(1,16)_ = 24.79, *p* = 1.36 × 10^−4^***	*F*_(1,16)_ = 0.05, *p* = 8.21 × 10^−1^ n.s.

Additional data relating to these analyses are provided in Extended Data Tables 2-1–2-3.

Nonsignificant *p*-values are indicated by “n.s.,” *p*-values <0.05 are indicated by “*,” <0.01 by “**,” and <0.001 by “***.”

10.1523/ENEURO.0154-23.2023.t2-1 Table 2-1 Raw data including physiology measurements, MBH and pituitary qPCR fold changes in gene expression, and pituitary RNA-sequencing differential expression data generated using edgeR. Differential expression analyses include those relating to simultaneous comparison of all groups, age comparisons, and photoperiod comparisons. Download Table 2-1, XLS file.

10.1523/ENEURO.0154-23.2023.t2-2 Table 2-2 Functional pathway analyses comparing 8L birds with 16L birds using the Database for Annotation, Visualization and Integrated Discovery (DAVID, Sherman et al 2022). Categories and functional analysis terms have been reported alongside associated gene count, significance levels, and a total list of significantly differentially expressed genes within each category. Download Table 2-2, XLS file.

10.1523/ENEURO.0154-23.2023.t2-3 Table 2-3 Functional pathway analyses comparing juvenile birds with adult birds using the Database for Annotation, Visualization and Integrated Discovery (DAVID, Sherman et al 2022). Categories and functional analysis terms have been reported alongside associated gene count, significance levels, and a total list of significantly differentially expressed genes within each category. Download Table 2-3, XLS file.

### Age-dependent photoperiodic changes in hypothalamic deiodinase expression

Two-way ANOVA identified a significant photoperiod by age interaction for hypothalamic *DIO2* expression (Fig. 1*c*, Table 2). Overall, there was no significant main effect of photoperiod on *DIO2* expression. There was a significant effect of age on *DIO2* expression. There was a significant interaction for hypothalamic *DIO3* expression (Fig. 1*d*). There was also a significant main effect of photoperiod and age. These findings suggest that *DIO2* and *DIO3* are highly sensitive to photoperiodic state in adult quail with significantly higher levels in long photoperiod and short photoperiod, respectively. Surprisingly, *DIO2* was observed to be significantly elevated in short photoperiods in juvenile birds. *DIO3* expression levels were similar in both long and short photoperiod conditions.

### Photoperiod- or developmental-induced changes in transcript expression in the pituitary gland

**Figure 2.**
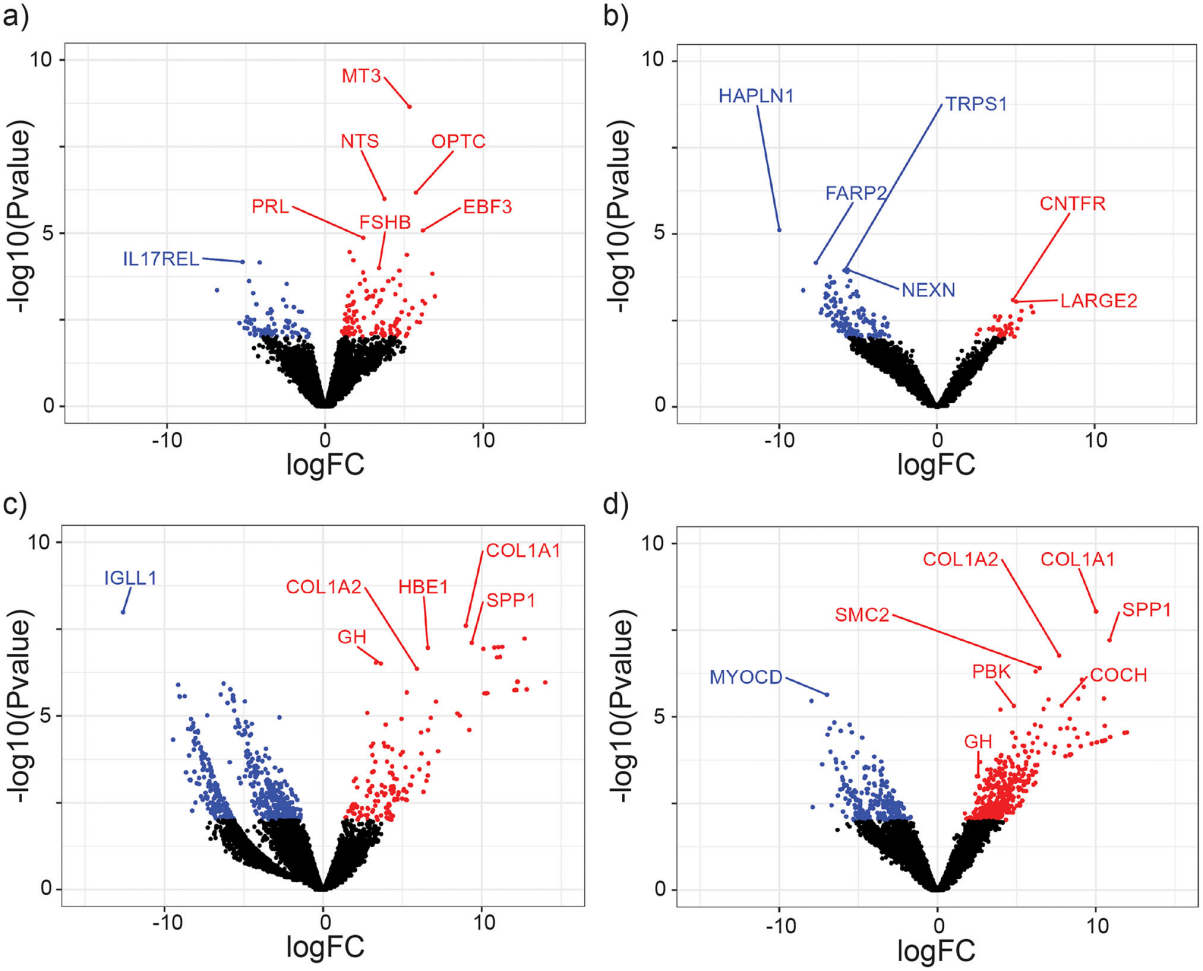
Volcano plots comparing age and photoperiod revealed a high number of differentially expressed transcripts. Upregulated transcripts (logFC > 1) are colored red, and downregulated transcripts (logFC < −1) are colored blue. ***a***, Significant differentially expressed transcripts across photoperiod treatments in adults (*n* = 206) included *FSHß*, *PRL*, and *NTS.* ***b***, Significant differentially expressed transcripts across photoperiod treatments in juveniles (*n* = 184) included *HAPLN1.* ***c***, Significant differentially expressed transcripts across 16L age groups (*n* = 685) included *GH*. ***d***, Significant differentially expressed transcripts across 8L age groups (*n* = 678) also included *GH.* A *p*-value of <0.01 was deemed significant for all plots.

**Figure 3.**
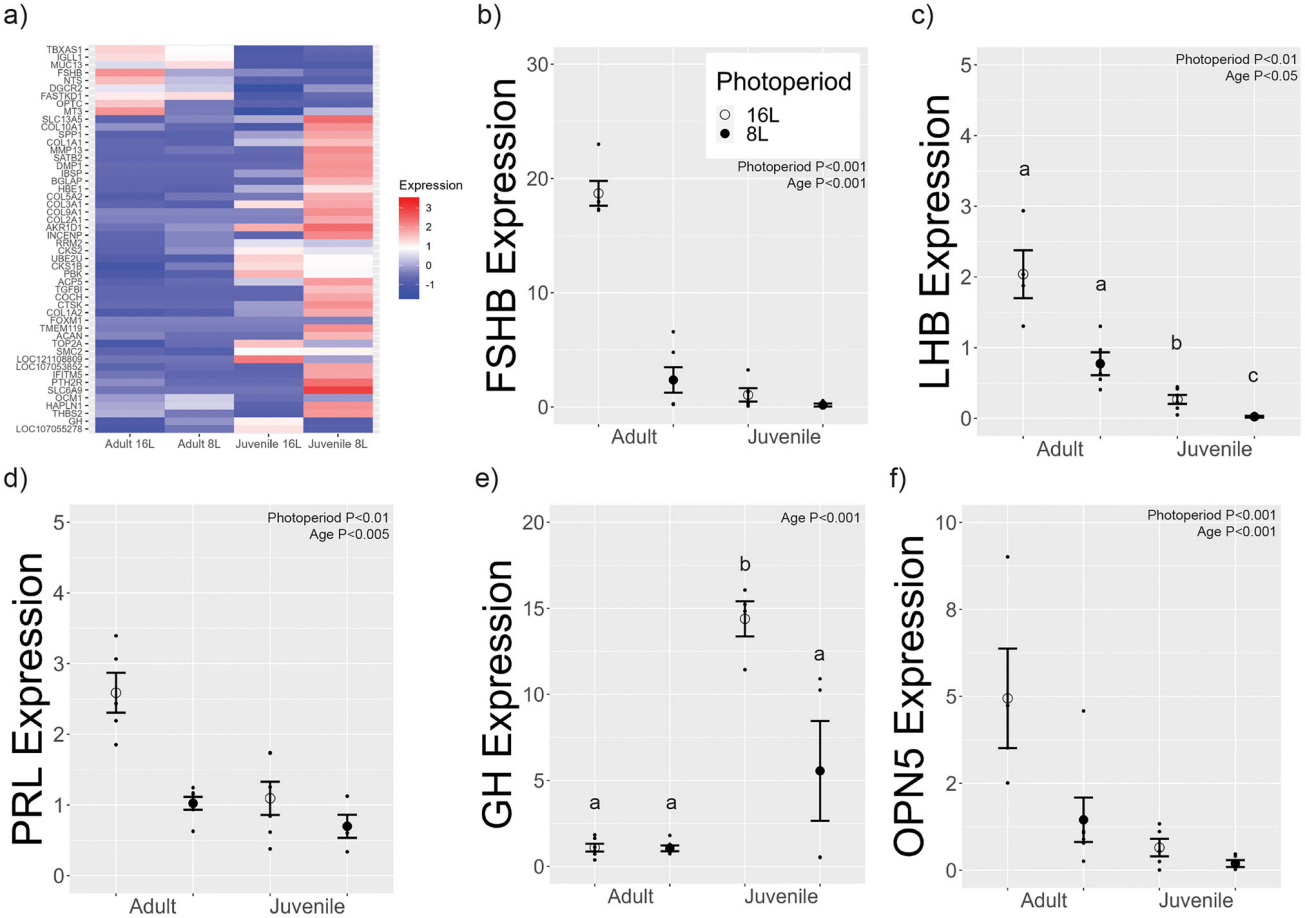
***a***, A heatmap of the top 50 most significant transcripts in the pituitary gland. Among the most significant transcripts were *FSHß* and *GH*. ***b–f***, Targeted qRT-PCR analyses were performed in order to investigate the transcription of genes of interest influenced by photoperiod and age. ***b–d***, There were significant main effects of both photoperiod and age on *FSHß*, *LHß*, and *PRL* expression, as well as an interaction effect between photoperiod and age on *LHß* expression. ***e***, There was a significant photoperiod by age interaction on *GH* expression. ***f***, There was a significant main effect of photoperiod and age on *OPN5* expression. For the pituitary sequencing heatmap analysis, a *p*-value of 0.01 was deemed significant. Targeted qRT-PCR analyses were performed using two-way ANOVA and Tukey's HSD with an *α* value of *p* = 0.05. The letters above each group indicate pairwise comparisons by Tukey's HSD where appropriate. Additional data relating to these analyses are provided in Extended Data Figures 3-1 and 3-2.

Our sequencing assays resulted in transcriptomes being produced with an average N50 value of approximately 1 kb and an average depth of 4.53. edgeR analyses identified 206 transcripts that were differentially expressed between long and short photoperiod treatments in adults (Fig. 2*a*; Extended Data Table 2-1). A total of 126 transcripts were upregulated, and 80 were downregulated in long compared with short photoperiod. As anticipated, *FSHß*, prolactin (*PRL*), and neurotensin (*NTS*) were found to be differentially expressed between long and short photoperiods in adults. A total of 184 transcripts were differentially expressed between long and short photoperiod treatments in juveniles (Fig. 2*b*). Forty transcripts were upregulated, and 144 were downregulated in long compared with short photoperiod. *HAPLN1* was found to be differentially expressed between long and short photoperiods in juveniles. A total of 685 transcripts were differentially expressed between 16 L adults and 16 L juveniles (Fig. 2*c*). A total of 175 transcripts were upregulated, and 510 were downregulated. *GH* was found to be differentially expressed between 16 L adults and 16 L juveniles. A total of 678 transcripts were differentially expressed between 8 L adults and 8 L juveniles (Fig. 2*d*). A total of 422 transcripts were upregulated, and 156 were downregulated. Again, *GH* was identified as a differentially expressed transcripts between 8 L adults and 8 L juveniles. edgeR identified that 331 transcripts were differentially expressed across the four treatment groups (Fig. 3*a*). A heatmap of the top 50 most significantly differentially expressed genes comparing across all four groups (adult and juvenile 8 L and 16 L) was plotted, including *FSHß* and *GH* (Fig. 3*a*). *PRL*, *FSHß*, *GH*, *OPN5*, *NTS*, *MEF2A*, and *MEF2D* were selected to plot the counts per million for each treatment group (Extended Data Fig. 3-1*A–G*). Relevant statistics have been provided in the appropriate figure legend.

10.1523/ENEURO.0154-23.2023.f3-1 Figure 3-1 Plots comparing transcript count data from Oxford Nanopore RNA-sequencing. A-C. *PRL*, *FSHß*, and *GH* were significantly differentially expressed across all groups. D. *OPN5* was not found to be differentially expressed, contrasting qPCR data. E. *NTS* was significantly differentially expressed, whereas F-G. *MEF2A* and *MEF2D* were not significantly differentially expressed across treatment groups. For all analyses, a P-value of P<0.01 was deemed significant. Download Figure 3-1, TIF file.

10.1523/ENEURO.0154-23.2023.f3-2 Figure 3-2 Schematic diagram representing photoperiod and developmental changes in pituitary cell type transcript expression. Expression of *FSHß*, *LHß*, *PRL*, *TSHß* and *GH* in pituitary cells is dependent on both the age of the quail and the experienced photoperiod conditions. Blank cells are included to indicate that transcriptomic changes are occurring in existing cells, rather than e.g., as a result of the production of new cells. Download Figure 3-2, TIF file.

### qRT-PCR analyses confirm transcript expression identified in transcriptome analyses

In order to confirm the differential expression, qRT-PCR analyses were performed on select neuroendocrine transcripts of interest including *FSHß*, *LHß*, *PRL*, *GH*, and *OPN5*. There was a significant main effect of photoperiod (Fig. 3*b*, Table 2) and age for *FSHß* expression. There was no significant interaction on *FSHß* expression. These data support the conjecture that *FSHß* expression is elevated in long photoperiods and is higher in adults. There was a significant photoperiod–age interaction on *LHß* expression (Fig. 3*c*). There were significant main effects for photoperiod and age on *LHß* expression. There was a significant main effect of photoperiod on *PRL* expression (Fig. 3*d*) and age. There was no significant photoperiod by age interaction on *PRL* expression. There was a significant photoperiod by age interaction on *GH* expression (*F*_(1,16) _= 5.67, *p* < 0.05; Fig. 3*e*). There was a significant main effect of age but not photoperiod on *GH* expression. There was a significant main effect of photoperiod on *OPN5* expression (Fig. 3*f*) and age. There was no significant photoperiod by age interaction. Overall, transcript analyses using qRT-PCR assay were consistent with the counts per million determined by transcriptome sequencing.

### Functional pathways

In order to determine the general functions of significantly differentially expressed transcripts of interest across photoperiod and age, DAVID functional annotation was used (Extended Data Tables 2-2, 2-3, respectively). For these analyses, a *p*-value of <0.05 was used as the significance cutoff. In the comparison between adults and juveniles, the functional groups with the highest significance included those involved in the extracellular matrix (ECM), ECM–receptor interaction, secretion, and focal adhesion. The photoperiod comparison again showed these functional groups as significant and also showed significant transcripts involved in symport, behavior, neuroactive ligand–receptor interaction, and transcription regulation.

## Discussion

This study demonstrates that somatotrophs significantly increase *GH* expression during development regardless of the prevailing photoperiod condition. Conversely, lactotrophs and gonadotrophs are primarily sensitive to photoperiod condition with increased *FSHß* and *LHß* expression in long days. Only *LHß* expression was dependent on both photoperiod and developmental condition suggesting this transcript has seasonal- and age-dependent regulation. Despite both adults and juveniles having smaller testes in short photoperiods, there are several transcripts that show developmental- and photoperiod-dependent expression. For example, juvenile quail had 143/144 transcripts upregulated in short photoperiod that were not significantly differentially expressed in adults. Moreover, there were 39/40 transcripts downregulated in juvenile quail without any significant differential expression in adults. Surprisingly, the well-characterized *DIO2* and *DIO3* photoperiodic changes in expression were only identified in adult birds. qRT-PCR analyses confirmed the gene count data identified by RNA sequencing and provided strong internal replication. Overall, these findings indicate a suite of photoperiod-induced molecular changes in the pituitary gland that are age-dependent and a plethora of targets that could help uncover how juveniles have a heightened sensitivity to photoperiod cues compared with adult birds.

### Long days facilitate gonadal growth in adults and juveniles

Both adults and juveniles had larger testes in a long photoperiod as opposed to a short photoperiod, consistent with previous publications (Robinson and Follett, 1982). Follett and Farner have previously shown that prolonged exposure of juvenile quail from hatching to long photoperiods can cause increased testis growth (Follett and Farner, 1966). The change in juveniles could be due to two potential mechanisms. First, similar to adults, the photoperiod induces light-dependent changes in gonadal growth. Alternatively, the second mechanism could be due to developmental programs. In hamsters, early exposure to photoperiod or melatonin can delay gonadal growth (Prendergast et al., 2013; Sáenz de Miera et al., 2017). In the current study, it is possible that short photoperiod exposure delayed the developmental program (or puberty) in juvenile quails. This conjecture also applies to the photoperiod-induced changes in fat score. Future analyses of the MBH and pituitary are required to establish the mechanisms upstream of the pituitary that influence photoperiodic and developmental programs in reproduction physiology.

### Adult neuroendocrine transcription in the MBH is typical for a long day response

Adult MBH *DIO2* and *DIO3* were increased and decreased in 16 L compared with 8 L respectively, as expected (Yasuo et al., 2005, 2006). We found that juvenile *DIO2* expression increased during short days and that *DIO3* expression did not significantly differ between long and short photoperiods. Given the role of *DIO2* and *DIO3* in the neuroendocrine signal transduction cascade that links photoreception with reproductive physiology (Nakane and Yoshimura, 2010), the dynamics of *DIO2* are surprising. These data appear to suggest that other molecular neuroendocrine changes drive photoperiodic sensitivity in juvenile quail. In juvenile hamsters, *DIO2* has similar levels in long and short photoperiod conditions (Prendergast et al., 2013). *DIO2* expression, leading to a hypothalamic increase in T3, is thought to establish reproductive maturation in mammals. *DIO2* has a wide role in development and has established roles for fisheye metamorphosis and in T3-dependent amphibian metamorphosis (Duarte-Guterman et al., 2010; Itoh et al., 2010). These data indicate that juvenile photoperiodic responsiveness might not be driven by tanycyte rewriting consistent with adult responses. An alternative proposition is other neuronal circuits associated with developmental control of the GnRH system are involved. The stimulation of pituitary cells by GnRH to secrete *LHß* and *FSHß* is partly regulated by the secretion of melatonin in sheep (Misztal et al., 2002). GABA also regulates GnRH neurons independent of the neuroendocrine response to changing photoperiod (Bentley et al., 2006). Hence, these findings support an alternative means by which pituitary *LHß* secretion may be governed outside of the *DIO2/DIO3*-involved neuroendocrine cascade.

### Photoperiod- and age-dependent changes in pituitary gland transcriptomes

Our volcano plot comparing differential expression of adults in long and short photoperiodic conditions revealed a total of 206 differentially expressed transcripts. A total of 126 transcripts were upregulated in a long photoperiod, and 80 were downregulated. Among the differentially expressed transcripts were *PRL*, *FSHß*, and *NTS*. Long photoperiod increases in *PRL* expression, associated with seasonal timing, are common within the literature (Goldsmith and Hall, 1980; Sharp, 2005). Similarly, *FSHß* content increases during long photoperiod conditions (Follett and Maung, 1978; Urbanski and Follett, 1982; Nicholls et al., 1983) where increased FSH in the pituitary gland precedes increases in plasma concentration. Given its role in acting directly on gonads to induce growth, it is not surprising that *FSHβ* levels should be high in stimulatory long day photoperiods. *NTS* has a suggested role in regulating *LH* secretion (Rostène and Alexander, 1997). Yamada and Mikami described the distribution of *LH*-releasing hormone (*LHRH*) as coinciding with dense populations of *NTS* when using comparable data between ducks and quail (Yamada and Mikami, 1981). Although *LHß* is absent from the reference *Coturnix japonica* reference genome, and therefore was not identified through RNA sequencing, *LHß* is known to increase in long photoperiod conditions (Follett et al., 1975; Nicholls et al., 1983). Hence, an accompanying increase in *NTS* expression would be expected.

Our volcano plot comparing differential expression across ages in 16 L birds revealed a total of 685 differentially expressed transcripts. A total of 175 transcripts were upregulated in juveniles, and 510 were downregulated. Critically, among the differentially expressed transcripts was *GH*, whose expression is known to be downregulated in adults (Schew et al., 1996). *GH* expression is therefore an excellent marker of age, supporting the significance of the remaining age-dependent differentially expressed transcripts presented here. Similar results were observed in our volcano plot comparing differential expression across ages in 8 L birds. Here, 678 transcripts were differentially expressed, with 422 being upregulated and 156 being downregulated in juveniles. Again, *GH* was one transcript whose upregulation in juveniles was apparent.

### Function pathways modified during photoperiod and developmental programming

A functional annotation analysis across photoperiodic conditions suggests an influence of seasonal time on transcripts involved with the brain and nervous system, as neuroactive ligand–receptor interaction scored highly in significance. In both neuroactive ligand–receptor interaction and secretion, *PRL* was identified as a key gene of interest. *FSHß* was also implicated in neuroactive ligand–receptor interaction. Therefore, this analysis confirms the photoperiodic differential expression of these important transcripts that we have focussed on in this manuscript.

The ECM–receptor interaction was identified as the most significant category in our comparison of photoperiodic gene functions. The ECM–receptor interaction is known to play an important role in tissue and organ morphogenesis, including adipogenesis (San et al., 2021). Furthermore, a recent study on the gonadal development of male geese has implicated significant enrichment of the ECM–receptor interaction pathway by differential gene expression in the pituitary gland (Tang et al., 2022). Therefore, the ECM–receptor interaction pathway is a likely area for the identification of photoperiodically significant transcripts. For example, integrin alpha 3 (*ITGA3*) showed significant differential expression within the ECM–receptor interaction pathway and is associated with neural migration. Within this pathway we also identified chondroadherin (*CHAD*), which has a role in mediating the adhesion of chondrocytes; and *COL2A1*, for the production of the pro-alpha1 chain of type-II collagen protein, suggesting that cartilage synthesis is an important physiological change occurring across photoperiodic time.

DAVID functional annotation analyses confirmed that many transcripts, particularly those implicated in growth and development, were differentially expressed across age groups. Transcripts involved in the ECM and ECM–receptor interaction were among the most significant in the functional annotation analysis. The ECM is critical for practically all tissue morphogenesis (Gullberg and Ekblom, 2003), hence why transcripts implicated in its expression are differentially expressed during the rapidly developing juvenile life stage. Furthermore, the TGF-ß signaling pathway was implicated as significant in this analysis. This pathway is thought to be involved in the testicular development of broiler roosters under long (16 L:8 D) photoperiod conditions (Sun et al., 2020) and functions similarly in quail (Otake and Park, 2016).

Similar to the photoperiod comparison, many named transcripts, implicated in the ECM–receptor interaction pathway, were identified in this comparison across age, including *CHAD* and *COL1A2*. Bone morphogenetic proteins 4, 5, and 6 (*BMP4*, *5*, and *6*) were implicated in secretion and differentially expressed between adults and juveniles. It is suggested that *BMPs* may have a role in the multiple craniofacial bone growth of birds; *BMP4* is critical for beak development and is expressed in multiple craniofacial bones of Huiyang bearded chickens, and is therefore likely an important transcript of interest when examining age differences in growth and development of Japanese quail (Hong et al., 2019).

Overall, it is apparent that these functional analyses provide a valuable resource for the identification of novel transcripts associated with both age and photoperiodism. Other groups in the field have used this common method of functional annotation similarly, in order to identify salient areas for future targeted research, for example, in migratory black-headed buntings (Sharma et al., 2018) and Japanese quail (Marasco et al., 2016).

### Targeted qRT-PCRs replicate findings obtained through transcriptome analyses

qRT-PCRs for *PRL*, *FSHß*, *LHß*, and *GH* confirm data generated using Oxford Nanopore RNA sequencing. *PRL* is a well-known marker of long photoperiod in birds (Yasuo et al., 2004; Sharp, 2005). qRT-PCR analyses indicate that *PRL* expression is significantly high in the adult 16 L group compared with the 8 L group. Surprisingly, there was a photoperiodic difference in juveniles. In juveniles, the lack of *PRL* change supports the conjecture that light exposure modifies a developmental program similar to the lack of changes observed in the MBH *DIO2/DIO3* system. Similarly, *FSHß* was increased in adults exposed to long photoperiods, consistent with previous reports (Yasuo et al., 2006). However, there was no change observed in the juvenile pituitary gland. *FSHß* causes gonadal cell proliferation and the inhibition of *FSHß* in quail results in the regression of testis size (Brown and Follett, 1977). The lack of a change in *FSHß* in the juveniles suggests that short photoperiod delayed testicular growth. We propose that *PRL* and *FSHß* are molecular markers of photoperiodic programs in adult Japanese quail.

*LHß* expression, however, was found to increase in both adult and juvenile 16 L conditions (Follett et al., 1975; Nicholls et al., 1983). It is interesting that *LHß* expression is increased in juveniles housed in long photoperiod and indicates that the testicular growth observed was a result of the direct action of *LHß* on the testes. These data indicate that *LHß* expression is under the control of photoperiodic programs in both adult and juvenile quail. It is currently unclear how gonadotrophs that express *LHß* and *FSHß* are differentially regulated by photoperiod and developmental programs, respectively. *OPN5* expression mirrored *LHß* in both adult and juvenile pituitary glands, suggesting a potential molecular mechanism that links light and *LHß* expression, independent of *FSHß* expression.

A clear example of a molecular marker of developmental programs is *GH* expression occurring in somatotrophs. In juvenile quail, there was a robust increase in *GH* expression, independent of photoperiod treatment. Conversely, *GH* expression was found to be consistent across photoperiod conditions in adults. For adults this is expected, as *GH* expression is no longer required for growth and development once maximum size has been reached, and hence ceases to be expressed in older birds (Scanes and Lauterio, 1984). One limitation of the current experiment is the inability to link *GH* expression with body mass.

## Conclusions and future research

Overall, the findings reported here indicate pituitary gland cell specificity for photoperiodic and developmental programs of the reproductive physiology of birds. The data indicate somatotrophs as a cellular basis of developmental programs, lactotrophs as a marker of photoperiod programs, and gonadotrophs as a mix of both programs (Extended Data Fig. 3-2); this conclusion builds on recently published work indicating a pituitary cell autonomy, separate from changes driven by the MBH, in seasonal *FSHß* expression (Majumdar et al. 2023). Our approach to conducting Oxford Nanopore RNA sequencing and qRT-PCR analyses to investigate the photoperiodic and developmental responses in quail provides a robust approach to provide a comprehensive and confirmatory strategy. The data generated suggested a wide range of potentially interesting transcripts in the comparisons between photoperiod and age, acting as a resource to guide future research. It is unclear which transcripts and gene ontology pathway analyses are associated with the pituitary cell types. The next steps are to establish which functional pathways are associated with photoperiodic and developmental programs at a cellular resolution. Moreover, many studies have highlighted that photoperiodic cues may be insufficient for full ovarian development indicating that other supplementary cues are required for full reproductive competence (Ball and Ketterson, 2008; Tolla and Stevenson, 2020). Sex differences in the adult and juvenile seasonal programs responses are therefore a salient avenue for future research.

## Synthesis

Reviewing Editor: Julie Bakker, University of Liege Decisions are customarily a result of the Reviewing Editor and the peer reviewers coming together and discussing their recommendations until a consensus is reached. When revisions are invited, a fact-based synthesis statement explaining their decision and outlining what is needed to prepare a revision will be listed below. The following reviewer(s) agreed to reveal their identity: NONE.

synthesis: 2 reviewers have seen your revision and both agree that the ms has been improved significantly. Some minor comments remain, please follow closely the comments and suggestions made by the reviewers.

Reviewer 1:

The authors have provided very detailed and exhaustive responses to the comments that had been presented by both reviewers. All responses appear logical and satisfactory.

The additional information that had been requested has been provided.

One could still argue about a couple of the decisions made but since the potential limitations are clearly presented in the paper this would not be constructive and the paper should, in my opinion, now be accepted for publication.

The first submission of this paper was raising a lot of questions and I had focused on these problems in my review. Now that all these questions have disappeared and that the nature of data is completely clear, I could precisely focus on how they are presented. A careful reading of the revised manuscript revealed a number of relatively minor problems in presentation and I am suggesting a number of potential improvements below.

Line 28 (Statements): the identified novel transcript correlated (are associated) with photoperiod-and developmental program but I do not think the authors have information to demonstrate that the transcript DRIVE thee programs

Lines 186-209: the statistical methods as explained in the text (methods and results sections) do not match what is said in the legend of figure 1 (2 way ANOVA vs. t-test for testes and fat score; 1 vs. 2 way ANOVA for qPCR). Also phrasing of text is sometimes inadequate: e.g., "DiO2 expression has a significant effect of age...." NO: age has a significant effect on DiO2. Presentation is also redundant. Some results are in the text and replicated in part in the legend of the figure. The statistical results should be explained in the text, not split between text and figure legends.

The phrasing of these 2 sections should be carefully edited and made coherent with the legend of figure 1.

The section on qPCR (lines 232-249) is hard to read since this is a listing of main effects or interactions with associated probabilities. A table summarizing these data (2 main effects and the interaction , with the associated F and P) associated with a few comments would be a lot easier. You could put all results in a table and then for each main effect and for the interaction just list the variables for which there is a significant effect.

And, this long list of probabilities is duplicated between the legend of figure 3 and the text, result section. This information should be in the text and not reproduced in the figure legend.

The way statistical results are reported in figures 1 and 3 is in my opinion inadequate. Letters as plotted here can be used for post-hoc tests following a significant interaction. If there is an effect of one or the other main factor, no post hoc test needs to be performed since these factor sonly have 2 levels and graphically significant effects can only be indicated by having brackets joining pairs of data points and adding an asterisk above the bracket. This might actually be difficult if the two main factors are associated with significant effects. Figures should be modified to comply.

Reviewer 2:

This article is a revised version of a previous research paper on age-dependent photoperiodism using Japanese quail as a model. The manuscript has much improved and the results and discussion provide more information and a clearer picture. The information presented here are definitely of interest and provide a very interesting framework to work with. The authors have answered to most of my comments and concerns. I'd still temper the conclusion regarding the impact of the interaction of development and photoperiod as the adult group was exposed to long day for 8 weeks compared to 10 days in juveniles. I do understand that looking at juvenile stage in these birds is definitely restricted by the time but still, one might argue that it is the 8 weeks long exposure to long day that has an impact, not the long days in adult. A better comparison would have been to look for a 5 days exposure to long day in adult. In addition, providing information for females, as required by most funding agencies, would have been interesting

Minor comments.

-Unless I missed it, the letters in the figure to indicate statistically significant differences are not reported in the legend and I am kind of surprised that in figure 1C, Juvenile in short day are different from the groups of adults, is that correct?

- line 191: 2 females had large ovaries... but no follicular development? This could be added

-line 210: I'd add transcript expression in the pituitary for clarity

- line 389: light exposure modifies a development program similar to a lack of change... Again, maybe I misinterpret something but for my point of view, that would be the reverse (if I forget about the duration of exposure to long day in juvenile and adult). Long days seems to work only in adult to increase FSH expression, while juvenile did not reach the stage when they can be receptive to long day... no? Similarly, for LHb, the authors (line 401) indicate that its expression is under the control of photoperiod. Yes, to some extent, but the fold increase is much higher (with significant difference) in adult
