# Supplementary figures and images for: Dissociating Mechanisms That Underlie Seasonal and Developmental Programs for the Neuroendocrine Control of Physiology in Birds
Source: eNeuro. 2024 Apr 8;11(4):ENEURO.0154-23.2023. doi: 10.1523/ENEURO.0154-23.2023 (PMC11007308; doi:10.1523/ENEURO.0154-23.2023)

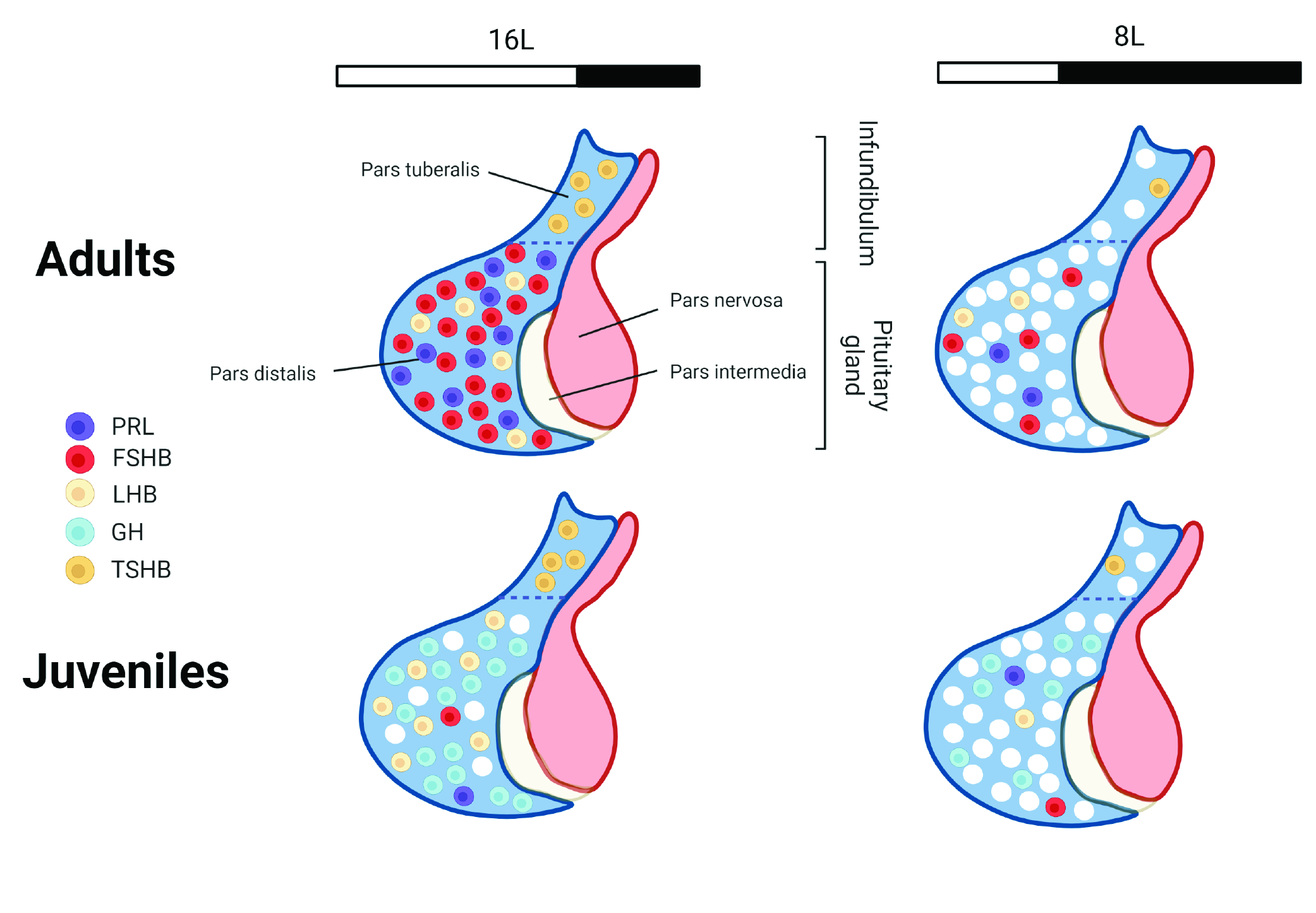

Supplement: Figure 3-2 — Schematic diagram representing photoperiod and developmental changes in pituitary cell type transcript expression. Expression of FSHß, LHß, PRL, TSHß and GH in pituitary cells is dependent on both the age of the quail and the experienced photoperiod conditions. Blank cells are included to indicate that transcriptomic changes are occurring in existing cells, rather than e.g., as a result of the production of new cells. Download Figure 3-2, TIF file. [file eneuro-11-ENEURO.0154-23.2023-s005.tif]
